# The Importance of Evaluating Primary Midwifery Care for Improving the Health of Women and Infants

**DOI:** 10.3389/fmed.2015.00017

**Published:** 2015-03-23

**Authors:** Ank de Jonge, Raymond de Vries, Antoine L. M. Lagro-Janssen, Address Malata, Eugene Declercq, Soo Downe, Eileen K. Hutton

**Affiliations:** ^1^Department of Midwifery Science, AVAG and the EMGO Institute for Health and Care Research, VU University Medical Center, Amsterdam, Netherlands; ^2^Center for Bioethics and Social Sciences in Medicine, School of Medicine, University of Michigan, Ann Arbor, MI, USA; ^3^Midwifery Academy Maastricht, Maastricht, Netherlands; ^4^Gender and Women’s Health Unit, Department of Primary and Community Care, Radboud University Medical Center, Nijmegen, Netherlands; ^5^Kamuzu College of Nursing, University of Malawi, Zomba, Malawi; ^6^Boston University School of Public Health, Boston, MA, USA; ^7^Research in Childbirth and Health (ReaCH) Group, School of Health, University of Central Lancashire, Preston, UK; ^8^Midwifery Education Program, Faculty of Health Sciences, McMaster University, Hamilton, ON, Canada

**Keywords:** primary health care, midwifery, medical intervention, maternal health services, newborn health

## Abstract

In most countries, maternal and newborn care is fragmented and focused on identification and treatment of pathology that affects only the minority of women and babies. Recently, a framework for quality maternal and newborn care was developed, which encourages a system-level shift to provide skilled care for all. This care includes preventive and supportive care that works to strengthen women’s capabilities and focuses on promotion of normal reproductive processes while ensuring access to emergency treatment when needed. Midwifery care is pivotal in this framework, which contains several elements that resonate with the main dimensions of primary care. Primary health care is the first level of contact with the health system where most of the population’s curative and preventive health needs can be fulfilled as close as possible to where people live and work. In this paper, we argue that midwifery as described in the framework requires the application of a primary care philosophy for all childbearing women and infants. Evaluation of the implementation of the framework should therefore include tools to monitor the performance of primary midwifery care.

## Primary Health Care

Since the Alma Ata conference in 1978, the WHO has advocated primary health care as the key to improving health and reducing health inequalities (1). The Alma Ata declaration shifted the focus in health care from treating diseases to promoting physical, mental, and social well-being as a fundamental human right and is still a major inspiration for health care reforms today (1). “Primary care provides accessible, comprehensive care, in an ambulatory setting, to patients in their own context on a continuous basis, and coordinates the care processes of patients across the healthcare system …” (2).

The Alma Ata declaration emphasizes the need to strengthen people’s own capabilities. The focus is on preventive, supportive care, and interventions or specialist care should be used only for complicated problems. To function well, primary care needs to be sustained by integrated and mutually supportive referral systems (3).

It has been shown that health care systems with a strong focus on primary care provide better population health. In these systems, health is distributed more equitably and with a more efficient use of resources (4). This is equally true for low-, middle-, and high-income countries (4). Considering the benefits of primary care, it is surprising that it is taking so long to move the emphasis in health systems to primary care (1). However, the move toward primary health care involves a major paradigm shift from focusing on the disease to regarding the whole person and from organizing care around the specialist to the generalist (5). This shift will take a long time to be accepted universally because it will be resisted by those who believe fervently in the old paradigm and the scientific norms that underpin it (1).

A similar system-level shift in maternal and newborn care is called for by the authors of a recent Lancet article on midwifery, in order to improve quality of care while avoiding overuse of medical interventions (6).

## Too Much and Too Little Health Care for Women and Newborns

Two of the millennium development goals (MDG’s) for 2015 are to improve maternal health and to reduce child mortality (7, 8). Although maternal mortality has decreased worldwide by 1.3% per year since 1990, only 16 out of 188 countries are expected to reach the MDG of a 75% reduction by 2015 (7). Equally, under-5 child mortality has reduced on average by 3.6% per year since 2005 but only 45 countries are expected to reach the MDG of reducing this rate by two thirds (8). In addition, a lack of good quality care leads to physical and psychological morbidity among millions of women each year worldwide (6).

The problem of poor quality care is twofold: on one hand, mothers and infants suffer from a lack of and access to services, but on the other hand, overuse of interventions leads to iatrogenic harm and high economic costs. In the face of rising costs for medical care and the projected increasing demands for health care in the next decades, this misapplication of medical resources is not only reprehensible in terms of health outcomes but also rapidly becoming unsustainable. For example, rising health care costs often motivate governments to increase out-of-pocket contributions (9), which, in turn, prevent those who cannot afford the higher rates from seeking necessary health care, exacerbating health inequalities.

**Figure 1:**
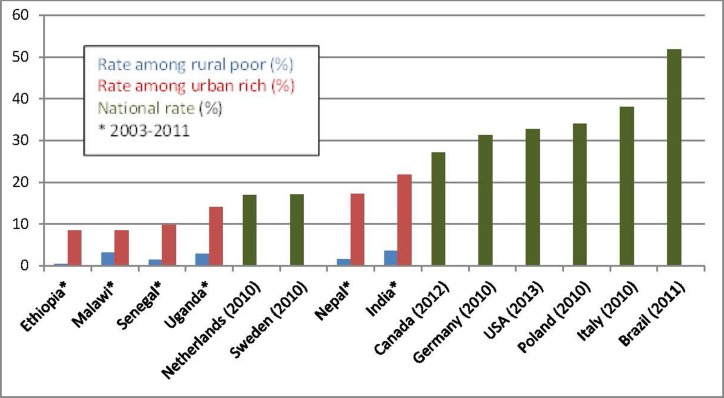
**Cesarean section rates in some selected countries (10–14)**.

Increasing rates of cesarean section (CS) offer an apt illustration of the increase in unnecessary medical treatments. There are huge variations in CS rates between countries and between regions within countries (see Figure 1) (10–14). In economically disadvantaged countries, CS rates are often too low among poor people and too high among people that are well off.

Avoidable CSs expose women and newborns to unnecessary risks of severe adverse outcomes and lead to unnecessary health care expenses of over US$ 2 billion a year worldwide (15). The rising CS rate is just one among several overused interventions throughout pregnancy, birth, and the postpartum period.

## Move toward Technology Driven, Specialist Maternity Care

For a long time, the prevailing paradigm in health has been the control of disease by interventions based on biomedical science (1). Technology facilitated the identification of risk factors for complications and interventions were used to prevent these complications from happening. However, these technologies and interventions were often used without evidence of their beneficial effect. Nevertheless, once technologies are part of routine care, their use becomes the norm. For example, electronic fetal monitoring is still routinely used, even though research shows that it does *not* improve neonatal outcomes among low risk women and it is associated with an *increase* in CSs (16). Overuse of interventions, intended to minimize risk, may actually contribute to a worsening of health outcomes. Although maternal mortality has decreased in many countries in the world since 1990, it is increasing in some high-income countries (7), where it may be associated with complications of unnecessary interventions (13).

The use of technologies in maternity care was facilitated by the move from home to hospital birth. Birth was redefined as an illness that required specialist care that could only be provided in hospitals (17). The move toward hospital birth occurred alongside the relocation of health care generally from home to hospital (17). It has been associated with a loss of autonomy for midwives. In some countries, midwives ceased to practice; in others, they continued to practice, but under the authority of medical staff (17). Evidence shows that health systems where midwives are absent have higher rates of unnecessary interventions and inequalities in care provision and outcomes (6). In such health systems rates of unnecessary elective CSs are high, which costs money that cannot be spent on primary care (6, 15).

The focus on technology and risk may be associated with fragmented care, often with little regard to women’s experiences of maternity care. In some countries, this fragmentation has led to consumer demands for more continuity of care, choice, and control (18, 19). In the United Kingdom, these demands culminated in a government report called *Changing Childbirth*, which had a considerable influence on national policy for many years (19).

## A System-Level Shift in Maternal and Newborn Care

**Figure 2:**
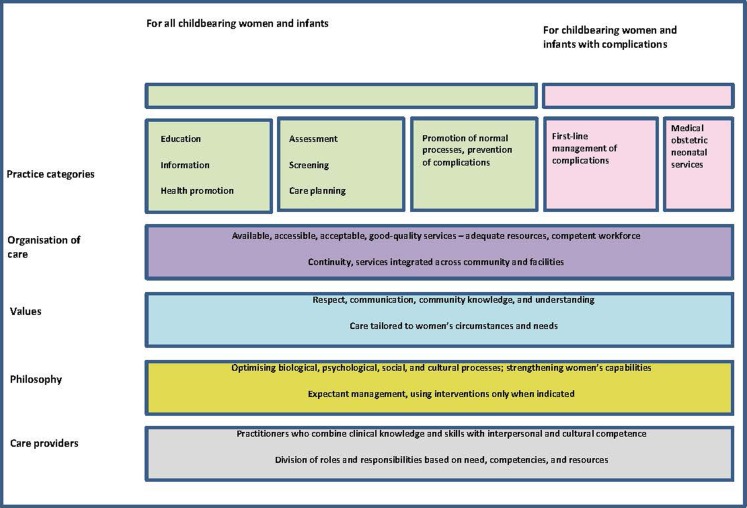
**The framework for quality maternal and newborn care (QMNC): maternal and newborn health components of a health system needed by childbearing women and their infants**. Reprinted from The Lancet, Vol. 384, Renfrew et al., Midwifery and quality care: findings from a new evidence-informed framework for maternal and newborn care, 129–45, Copyright 2014, with permission from Elsevier (6).

In order to reimagine the organization of maternity care, authors of a recent Lancet article, conducted systematic reviews of studies of women’s views and experiences of maternity care and the outcomes of that care (6). Based on their findings, the authors call for a “system-level shift from fragmented maternal and newborn care focused on identification and treatment of pathology for the minority to skilled care for all” (6). They emphasize the importance of preventive and supportive care that strengthens women’s capabilities and meets their needs. The focus should be on promotion of normal reproductive processes while access to management of complications and emergency treatment should be available when necessary (see Figure 2) (6).

The resulting quality maternal and newborn care (QMNC) framework was based on aspects of care that are important to women. These aspects are organized in five essential components of the framework: the characteristics of care providers, philosophy, values, organization of care, and effective practices.

Midwifery care is pivotal in this framework and is defined as “skilled, knowledgeable, and compassionate care for childbearing women, newborn infants …. Core characteristics include optimizing normal biological, psychological, social, and cultural processes of reproduction and early life; timely prevention and management of complications; consultation with and referral to other services; respect for women’s individual circumstances and views; and working in partnership with women to strengthen women’s own capabilities to care for themselves and their families” (6).

In many countries, aspects of midwifery care are provided by a range of different health workers such as midwives, obstetricians, family doctors, nurses, auxiliary midwives, and traditional birth attendants. However, there are indications that the best maternal and perinatal outcomes are achieved if midwifery care is provided by educated midwives who work in collaboration as part of interdisciplinary teams providing integrated care across community and hospital settings (6).

The QMNC framework contains several elements that are similar to dimensions of primary care. In fact, midwifery as described in the framework requires the application of a primary care philosophy for all childbearing women and infants, even if they need hospital care for complications. Implementation of the framework will necessitates the development of primary midwifery care, which is mainly delivered by midwives.

Definitions of primary care in general, are directly applicable to primary midwifery care. Primary midwifery care is the first level of contact with the maternity care system; it fulfils most preventive and curative maternal and newborn health needs as close as possible to where people live and work; it is the first element of a continuing health care process and coordinates the care processes of women and infants across the maternity care system (2, 3). Although primary midwifery care should be offered in the community setting wherever possible, the philosophy underpinning primary care (e.g., strengthening people’s own capabilities) should also apply to hospital care.

## The Evaluation of Primary Midwifery Care

To shift the emphasis in maternity care from specialist to primary care, it is important to have tools to evaluate the performance of primary midwifery care (2, 20). The Primary Health Care Activity Monitor for Europe (PHAMEU) project showed that a European country’s strength of primary care can be measured using three dimensions at the structural level and four at primary care service-delivery (2, 20). The structural level will not be discussed in this paper. The dimensions of primary care at the service-delivery level are 1) access to primary care services, 2) comprehensiveness of primary care services, 3) continuity of care, and 4) coordination of care. We briefly describe how the general dimensions of primary care service-delivery can be applied to primary midwifery care.

### Access to primary care maternal and newborn services

This dimension could be measured by the density of the midwifery workforce, geographic availability, access to the maternity care system, affordability of services, and women’s satisfaction (2, 20).

Ensuring access to primary care is more than increasing coverage of services. The services offered need to be respectful, woman-centered, and of high quality. Respectful care is an important quality indicator in itself and women will also avoid services if they expect not to be treated well (21).

Although many aspects of midwifery care may be most effectively provided in the community setting, hospital birth is the norm in industrialized countries and is advocated in others to improve maternal and neonatal outcomes (7). In some countries, such as the United Kingdom, Canada, and the Netherlands, low rates of maternal and neonatal risks have been found among planned home births in low risk women (22–24). However, these studies have been conducted in countries with good risk selection, transportation, and referral systems and may only apply to settings with such integrated systems.

Even if women do not give birth at home, access to primary care in maternity services can be offered in midwifery units in a hospital or in freestanding birth centers. A systematic review showed that when compared to conventional hospital labor wards, these alternative settings were associated with reduced rates of medical interventions and increased rates of spontaneous vaginal births, maternal satisfaction, and continued breastfeeding at 1–2 months after birth, while health outcomes were similar (25). In keeping with these research findings, the recent guideline on intrapartum care, published by the National Institute of Clinical Excellence (NICE) advises birth in a midwifery unit as particularly suitable for primiparous women and at home or in a midwifery unit for multiparous women (26).

### Comprehensiveness of primary care services

Comprehensiveness refers to the availability of a wide range of services for common needs, which is important because it prevents unnecessary referrals to specialist care (4). Comprehensiveness in maternal and newborn care could be evaluated by the role of the midwifery practitioner as the first contact of care, the availability of equipment and tests in primary care, follow up of women solely in primary care (in other words: not referred to specialist care), and the provision of preventive care and health promotion (2, 20).

Prenatal care is often, but not always, provided in primary care. However, many medical procedures such as prenatal screening or diagnosis for congenital abnormalities and ultrasound often take place in obstetric units. Moving resources to primary care settings for tests and procedures that are proven to be sufficiently sensitive and specific might limit the overuse of screening, diagnostic, and monitoring techniques, especially if primary care is organized on the basis of continuity of care models. This requires up-skilling primary care professionals in performing these tests and in counseling women about the advantages and disadvantages of such procedures (27).

The use of interventions during labor in primary care is more controversial (28). In Canada, midwives can induce labor for postdates and augment labor with oxytocin while women remain in midwife-led care (29). It could be argued that these interventions support physiological labor rather than treat complications and therefore should be part of primary care. On the other hand, the lure of technology is powerful and the benefits of primary care can be undermined if it becomes as technology reliant as hospital based care. Care should be taken to not allow these technologies to become the focus of primary care. Technologies should be used within the context of woman-centered, supportive, and preventive care and be limited to those that have proven to be beneficial.

### Continuity of care

Continuity of care consists of longitudinal continuity of care (continuous care throughout pregnancy, labor, and the postpartum period), relational continuity of care (quality of the longitudinal relationship), and informational continuity of care (a woman’s medical information being readily available to any health care provider caring for her) (2, 20, 30). Sometimes “management continuity of care” is added as well (20) but this overlaps with coordination of care. Continuity of care allows care to be person-focused over time and to be coordinated through information transfer and recognition of risk factors or complications that require referral to specialist services.

A recent systematic review showed that midwife-led continuity of care compared to shared care or medical-led models of care were associated with higher rates of spontaneous vaginal birth and being attended by a known midwife, a longer mean length of labor and lower rates of instrumental birth, episiotomy, regional anesthesia, preterm birth, and fetal loss below 24 weeks (30). The rate of overall fetal and neonatal death between groups was similar. Midwife-led care was also associated with higher rates of maternal satisfaction and a trend toward lower costs.

### Coordination of care

Coordination of care could be evaluated by the presence of an effective gate keeping system (referral by a primary care professional to specialist care, so that specialist interventions are only used for those who need them), skill mix (optimum mix of different types of primary care providers), seamless collaboration between sectors (for example, through outreach of specialist care in primary care), and public health integration (use of clinical data to identify local health needs or priorities for health policy) (2, 20).

Primary midwifery care professionals have a higher chance of getting to know a woman within the context of her daily life and will be able to connect with other sectors such as welfare and housing to encourage health in the broadest sense of the word. Primary care also facilitates supporting women with complex social and psychological problems, for example, as a result of family violence, sexual abuse, health illiteracy, or migration difficulties. In addition, health promotion can be offered more effectively within the context of where women live and raise their children. Based on their observations in the community, midwives can be advocates for women and urge policy makers to address important social problems.

Good multidisciplinary collaboration is vital, not only within primary care but also between primary and specialist care. Primary midwifery care professionals should work in collaboration as part of interdisciplinary teams providing integrated care across community and hospital settings in an atmosphere characterized by respect for the unique skills of each member of the team (6).

## Conclusion

The prevailing paradigm in health care has been the control of disease by interventions based on biomedical science. Since the late 1970s, the WHO has been advocating a shift toward primary health care. A similar system-level shift is called for in the recently developed QMNC framework. We have argued that midwifery care requires the application of a primary care philosophy for all childbearing women and newborns and that this is enacted by the provision of primary care services by midwives. Evaluation of the implementation of the framework should therefore include tools to assess the performance of primary midwifery care. The monitor developed in the PHAMEU project consists of a set of indicators for primary care in general, which can be applied to midwifery in future studies.

## Conflict of Interest Statement

The authors declare that the research was conducted in the absence of any commercial or financial relationships that could be construed as a potential conflict of interest.

## References

[1] Bhatia M Rifkin SB. Primary health care, now and forever? A case study of a paradigm change. Int J Health Serv (2013) 43(3):459–71. 10.2190/HS.43.3.e 24066415

[2] Kringos D Boerma W Bourgueil Y Cartier T Dedeu T Hasvold T The strength of primary care in Europe: an international comparative study. Br J Gen Pract (2013) 63(616):e742–50. 10.3399/bjgp13X674422 24267857 PMC3809427

[3] Hixon AL Maskarinec GG. The Declaration of Alma Ata on its 30th anniversary: relevance for family medicine today. Fam Med (2008) 40(8):585–8. 18988046

[4] Starfield B. Primary care: an increasingly important contributor to effectiveness, equity, and efficiency of health services. SESPAS report 2012. Gac Sanit (2012) 26(Suppl 1):20–6. 10.1016/j.gaceta.2011.10.009 22265645

[5] Van Weel C Primary health care and family medicine at the core of health care: challenges and priorities in how to further strengthen their potential. Front Med (2014) 1:37 10.3389/fmed.2014.00037 PMC4292187 25593911

[6] Renfrew MJ McFadden A Bastos MH Campbell J Channon AA Cheung NF Midwifery and quality care: findings from a new evidence-informed framework for maternal and newborn care. Lancet (2014) 384(9948):1129–45. 10.1016/S0140-6736(14)60789-3 24965816

[7] Kassebaum NJ Bertozzi-Villa A Coggeshall MS Shackelford KA Steiner C Heuton KR Global, regional, and national levels and causes of maternal mortality during 1990-2013: a systematic analysis for the Global Burden of Disease Study 2013. Lancet (2014) 384(9947):980–1004 10.1016/S0140-6736(14)60696-6 24797575 PMC4255481

[8] Wang H Liddell CA Coates MM Mooney MD Levitz CE Schumacher AE Global, regional, and national levels of neonatal, infant, and under-5 mortality during 1990-2013: a systematic analysis for the Global Burden of Disease Study 2013. Lancet (2014) 384(9947):957–79. 10.1016/S0140-6736(14)60497-9 24797572 PMC4165626

[9] Lee WY Shaw I. The impact of out-of-pocket payments on health care inequity: the case of national health insurance in South Korea. Int J Environ Res Public Health (2014) 11(7):7304–18. 10.3390/ijerph110707304 25046630 PMC4113877

[10] Health Indicators 2012: Caesarean Section. Canadian Institute for Health Information (2012). Available from: www.cihi.ca

[11] Cavallaro FL Cresswell JA Franca GV Victora CG Barros AJ Ronsmans C. Trends in caesarean delivery by country and wealth quintile: cross-sectional surveys in southern Asia and sub-Saharan Africa. Bull World Health Organ (2013) 91(12):914–22. 10.2471/BLT.13.117598 24347730 PMC3845270

[12] Do Carmo Leal M Esteves Pereira AP Soares Madeira Domingues RM Theme Filha MM Bastos Dias MA Nakamura-Pereira M Obstetric interventions during labor and childbirth in Brazilian low-risk women. Cad Saúde Pública (2014) 30:S1–31. 10.1590/0102-311X00151513 25167177

[13] EURO-PERISTAT Project with SCPE and EUROCAT. European Perinatal Health Report. The Health and Care of Pregnant Women and Babies in Europe in 2010. EURO-PERISTAT (2013). Available from: www.europeristat.com

[14] Martin JA Hamilton BE Osterman MJK. Births in the United States, 2013. NCHS Data Brief, No. 175. Hyattsville, MD: National Center for Health Statistics (2014). 25483923

[15] Gibbons L Belizan JM Lauer JA Betran AP The Global Numbers and Costs of Additionally Needed and Unnecessary Caesarean Sections Performed Per Year: Overuse as a Barrier to Universal Coverage. Background Paper 30. World Health Report. Geneva: World Health Organization (2010).

[16] Alfirevic Z Devane D Gyte GML Continuous cardiotocography (CTG) as a form of electronic fetal monitoring (EFM) for fetal assessment during labour. Cochrane Database Syst Rev (2013) 5:CD006066 10.1002/14651858.CD006066.pub2 16856111

[17] Declercq E De Vries R Viisainen K Salvesen HB Wrede S Where to give birth? Politics and the place of birth. In: De Vries R Benoit C Van Teijlingen E Wrede S editors. Birth by Design. New York, NY: Routledge (2001). p. 7–27.

[18] Kennedy P. Healthcare reform: maternity service provision in Ireland. Health Policy (2010) 97(2–3):145–51. 10.1016/j.healthpol.2010.04.002 20483499

[19] Wrede S Benoit C Sandall J The state and birth/the state of birth: maternal health policy in three countries. In: De Vries R Benoit C Van Teijlingen E Wrede S, editors. Birth by Design. New York, NY: Routledge (2001). p. 28–50.

[20] Kringos DS Boerma WG Bourgueil Y Cartier T Hasvold T Hutchinson A The European primary care monitor: structure, process and outcome indicators. BMC Fam Pract (2010) 11:81. 10.1186/1471-2296-11-81 20979612 PMC2975652

[21] Freedman LP Kruk ME Disrespect and abuse of women in childbirth: challenging the global quality and accountability agendas. Lancet (2014) 384(9948):e42–4 10.1016/S0140-6736(14)60859-X 24965825

[22] Brocklehurst P Hardy P Hollowell J Linsell L Macfarlane A McCourt C Perinatal and maternal outcomes by planned place of birth for healthy women with low risk pregnancies: the Birthplace in England national prospective cohort study. BMJ (2011) 343:d7400. 10.1136/bmj.d7400 22117057 PMC3223531

[23] De Jonge A Geerts C van der Goes B Mol B Buitendijk S Nijhuis J. Perinatal mortality and morbidity up to 28 days after birth among 743 070 low-risk planned home and hospital births: a cohort study based on three merged national perinatal databases. BJOG (2014). 10.1111/1471-0528.13084 25204886

[24] Hutton EK Reitsma AH Kaufman K. Outcomes associated with planned home and planned hospital births in low-risk women attended by midwives in Ontario, Canada, 2003-2006: a retrospective cohort study. Birth (2009) 36(3):180–9. 10.1111/j.1523-536X.2009.00322.x 19747264

[25] Hodnett ED Downe S Walsh D Alternative versus conventional institutional settings for birth. Cochrane Database Syst Rev (2012) 8:CD000012 10.1002/14651858.CD000012.pub4 22895914 PMC7061256

[26] National Institute for Health and Care Excellence. Intrapartum Care. Care of Healthy Women and their Babies During Childbirth. (Vol. 190). London: NICE (2014). 25950072

[27] Gitsels-van der Wal JT Verhoeven PS Mannien J Martin L Reinders HS Spelten E Factors affecting the uptake of prenatal screening tests for congenital anomalies; a multicentre prospective cohort study. BMC Pregnancy Childbirth (2014) 14:264. 10.1186/1471-2393-14-264 25106057 PMC4137078

[28] Perdok H Mokkink L van DJ Westerneng M Jans S Mol BW Opinions of maternity care professionals about integration of care during labor for “moderate risk” indications: a Delphi study in the Netherlands. Birth (2014) 41(2):195–205. 10.1111/birt.12102 24702519

[29] Induction and Augmentation of Labour. Toronto, ON: College of Midwives of Ontario (2014).

[30] Sandall J Soltani H Gates S Shennan A Devane D Midwife-led continuity models versus other models of care for childbearing women. Cochrane Database Syst Rev (2013) 8:CD004667 10.1002/14651858.CD004667.pub3 23963739

